# Highlighting the evidence gap: how cost-effective are interventions to improve early childhood nutrition and development?

**DOI:** 10.1093/heapol/czu055

**Published:** 2014-06-23

**Authors:** Neha Batura, Zelee Hill, Hassan Haghparast-Bidgoli, Raghu Lingam, Timothy Colbourn, Sungwook Kim, Siham Sikander, Anni-Maria Pulkki-Brannstrom, Atif Rahman, Betty Kirkwood, Jolene Skordis-Worrall

**Affiliations:** ^1^Institute for Global Health, University College London, London WC1N 1EH, UK, ^2^Maternal and Child Health Intervention Research Group, London School of Hygiene and Tropical Medicine, London WC1E 7HT, UK, ^3^Human Development Research Foundation, Islamabad 44000, Pakistan, ^4^Epidemiology and Global Health, Umeä University, 90187 Umeå, Sweden, ^5^Institute of Psychology, Health & Society, Child Mental Health Unit, University of Liverpool, Liverpool L69 3BX, UK, ^6^Department of Population Health and Faculty of Epidemiology and Population Health, London School of Hygiene and Tropical Medicine, London WC1E 7HT, UK, ^7^Health Economics and Systems Group, London School of Hygiene and Tropical Medicine, London WC1E 7HT, UK and ^8^Health Economics Unit, University of Cape Town, Observatory 7925, South Africa

**Keywords:** Cost-effectiveness analysis, early childhood development, nutrition, review

## Abstract

There is growing evidence of the effectiveness of early childhood interventions to improve the growth and development of children. Although, historically, nutrition and stimulation interventions may have been delivered separately, they are increasingly being tested as a package of early childhood interventions that synergistically improve outcomes over the life course. However, implementation at scale is seldom possible without first considering the relative cost and cost-effectiveness of these interventions. An evidence gap in this area may deter large-scale implementation, particularly in low- and middle-income countries. We conduct a literature review to establish what is known about the cost-effectiveness of early childhood nutrition and development interventions. A set of predefined search terms and exclusion criteria standardized the search across five databases. The search identified 15 relevant articles. Of these, nine were from studies set in high-income countries and six in low- and middle-income countries. The articles either calculated the cost-effectiveness of nutrition-specific interventions (*n* = 8) aimed at improving child growth, or parenting interventions (stimulation) to improve early childhood development (*n* = 7). No articles estimated the cost-effectiveness of combined interventions. Comparing results within nutrition or stimulation interventions, or between nutrition and stimulation interventions was largely prevented by the variety of outcome measures used in these analyses. This article highlights the need for further evidence relevant to low- and middle-income countries. To facilitate comparison of cost-effectiveness between studies, and between contexts where appropriate, a move towards a common outcome measure such as the cost per disability-adjusted life years averted is advocated. Finally, given the increasing number of combined nutrition and stimulation interventions being tested, there is a significant need for evidence of cost-effectiveness for combined programmes. This too would be facilitated by the use of a common outcome measure able to pool the impact of both nutrition and stimulation activities.

**KEY MESSAGES**

- There is a scarcity of published literature on the cost-effectiveness of early childhood nutrition and development interventions. This may prevent the scale-up and replication of such interventions.
- There is a paucity of evidence from middle- and low-income countries and very few analyses are conducted from a societal perspective.
- Owing to the differences in outcome measures, it is difficult to compare the cost-effectiveness of these interventions.

## Introduction

The period from conception until the first 2 years of life is critical for the development of neural networks essential for perception and cognitive development (Walker *et al.* 2011). During this phase, children’s development is further affected by individual neurobiology, relationships with caregivers, and physical and psychosocial stimuli in the caregiving environment (Campbell and Ramey 1994; Grantham-McGregor *et al.* 2006; Walker *et al.* 2006b; Engle *et al.* 1997). Young children may be exposed to physical risks such as poor maternal nutrition, low-birthweight and infectious diseases, and psycho-social risks such as maternal depression, exposure to violence and lack of stimulation. Single or cumulative exposure to these risks can affect health and cognitive development over the life cycle (Schweinhart *et al.* 1986; Andersen *et al.* 2003).

The objective of interventions aimed at improving early childhood nutrition and development (ECND) is to reduce exposure to detrimental stimuli and provide children with an enabling environment (Van der Gaag and Tan 1998). Typically, interventions are targeted at poorer or more vulnerable groups and may include parenting and education support, complementary feeding, nutritional supplements and stimulation packages and activities (Grantham-McGregor *et al.* 1997; Leung *et al.*2003; Klein and Rye 2004; Jin *et al.* 2007; Dewey and Adu-Afarwuah 2008; Cooper *et al.* 2009; Ertem *et al.* 2006; Bentley *et al.* 2010; Aboud and Akhter 2011; Engle *et al.* 2011). Interventions may be delivered through home visits, community groups, clinic services and media campaigns (Walker *et al.* 2006b; Engle *et al.* 2011).

ECND interventions have a long history of success. One of the first studies demonstrating the impact of ECND was conducted in Kingston, Jamaica in 1986–89. That study examined the effects of nutritional supplementation, psychosocial stimulation or both, on the development of stunted children aged 9–24 months (Grantham-McGregor *et al.* 1991). In the short term, the development of children receiving nutrition supplementation was better than the control group. Children receiving both nutritional supplementation and stimulation had better development outcomes than those receiving only stimulation. Participating children were revisited at 7, 11 and 17 years of age. At 7 and 11 years, the children in the intervention arms had slightly higher test scores than the control group (Grantham-McGregor *et al.* 1997). At 17 years, children in the stimulation arm had higher psychosocial functioning. Children in the nutritional arm had small gains in height and energy intake compared with the control group. However, there were no significant interactions between stimulation and nutritional supplementation (Walker *et al.* 2006a).

This early intervention tested a combination of ECND components, i.e. nutritional supplementation and stimulation and demonstrates the short- and long-term benefits of ECND. Other interventions testing single or multiple components have also proven effective at improving ECND outcomes. Maulik and Darmstadt (2009) review 53 studies set in high-, middle- and low-income countries that focus on play, reading, music, stimulation and growth improvement interventions and find direct and indirect benefits for child development outcomes. Nores and Barnett (2010) review the effects of 30 cash transfer, nutritional, educational and combined interventions in Europe, Asia, Africa, Central and South America. Overall, they find positive effects on child development for all four categories of programmes. Engle *et al.* (2011) review 30 studies of ECND interventions set in low- and middle-income countries, particularly parenting and preschool enrolment. These studies showed evidence of their effectiveness in improving children’s cognitive, social and emotional development and school readiness.

Despite the availability of a large body of evidence on the success of ECND interventions in improving children’s outcomes, there is a paucity of economic evaluations of such interventions. Further, no comparable review of the economic evaluations of ECND interventions has yet been published.

Economic evaluations can range from total cost or cost of delivery studies that simply enumerate the cost of programme activities to more sophisticated cost-effectiveness analyses (CEA) that consider programme impact. Total cost or cost of delivery studies are an important starting point to inform resource allocation. However, without some sense of the scale of the intervention, or what that money was able to purchase, the policy implications would be unclear. CEA is an economic evaluation that compares the costs and outcomes of two or more courses of action (Edejer *et al.* 2003; Drummond *et al.* 2005; Batura *et al.* 2014). It is well established in the literature that a CEA can assist in allocating competing resources where they are likely to have the biggest effect (Johns *et al.* 2003; Berger and Teutsch 2005). CEAs can also directly inform decisions regarding the replication and scale-up of interventions (Johns *et al.* 2003; Drummond *et al.* 2005). Cost-effectiveness is usually expressed as a ratio where the denominator is an improvement in the health outcome, and the numerator is the cost associated with that improvement. The impact or effect is generally measured in non-monetary units that capture improvements in the quality or quantity of life such as years of life gained, infant deaths averted and cases of stunting averted (Walker *et al.* 2006b; Engle *et al.* 2011). As the CEA measures outcomes in non-monetary terms, it is more appropriate for the economic evaluation of ECND interventions than other forms of economic evaluation such as cost–benefit analysis, which measures outcomes in monetary terms (Drummond *et al.* 2005; Mogyorosy and Smith 2005). This is because it can be difficult, and sometimes, controversial to calculate a monetary value for children’s health and development (Cellini and Kee 2010; Dhaliwal *et al.* 2012). Composite measures can also be used to combine effects on mortality and morbidity and compare outcomes on the same scale. Quality-adjusted life years (QALYs) and disability-adjusted life years (DALYs) are the most commonly used composite measures (Edejer *et al.* 2005). Although costs are generally comparable across programme options, cost-effectiveness ratios are only directly comparable when the same measure of effect is used (Creese *et al.* 2002).

Thus, improvements in ECND mean that healthy children grow to become healthy adults (Smith and Haddad 2000), resulting in a smaller burden on health systems that are especially fragile in low- and middle-income countries. Governments often have competing priorities in resource-constrained settings. In such cases, the implementation, replication or scale-up of successful ECND interventions is seldom possible without comparing the cost-effectiveness of these interventions vs others that benefit children at this vulnerable stage. Decision makers need an understanding of associated costs and outcomes to make informed choices. This article aims to enhance that understanding of cost-effectiveness of interventions to improve ECND in low- and middle-income countries.

## Methods

The aim of this article is to summarize and critically review the available evidence on the cost-effectiveness of ECND interventions. Such a review of evidence also allows us to examine the methodology used to build the knowledge base, and to identify best practice for developing this knowledge base further.

Four databases were searched to identify relevant articles: Scopus, PubMed, Web of Science and the Cochrane Database of Reviews. In addition to these, we also searched Google Scholar and hand-searched the references of identified articles. The keywords used in the initial search strategy were: ‘cost effectiveness analysis’; ‘cost of delivery’; ‘early childhood development’; ‘nutrition’; ‘randomised control trials’; ‘RCTs’; ‘intervention programmes’; ‘under the age of 2’; ‘under the age of two’; ‘under 2-s’; ‘under twos’. Using this strategy, we found only a very small number of published articles on the total cost or cost-effectiveness of randomized control trials (RCTs) of ECND interventions (*n* = 4).

The search was then expanded to include the following additional keywords: ‘nutritional supplementation/ fortification’; ‘breastfeeding’; ‘diarrhoea’; ‘stimulation’; ‘parenting’; ‘day care/ crèche’; ‘preschool’. For those words that have alternate spellings, we included these in the search terms; for example, crèche and creche; preschool and pre-school; daycare and day care; diarrhea and diarrhoea. We included articles written in English and published in peer-reviewed journals between 1980 and 2012. We also included articles that performed a total cost analysis, cost-effectiveness or cost utility analysis of an RCT of an ECND intervention. We excluded articles that were trial protocols or systematic reviews. We also excluded articles that conducted economic evaluations of vaccination programmes or of preventive actions or therapies against bacterial or viral transmissions of disease. Further, we excluded articles where the study population was older than 5 years and the outcome was not related to ECND, for example, neonatal mortality.

**Figure 1:**
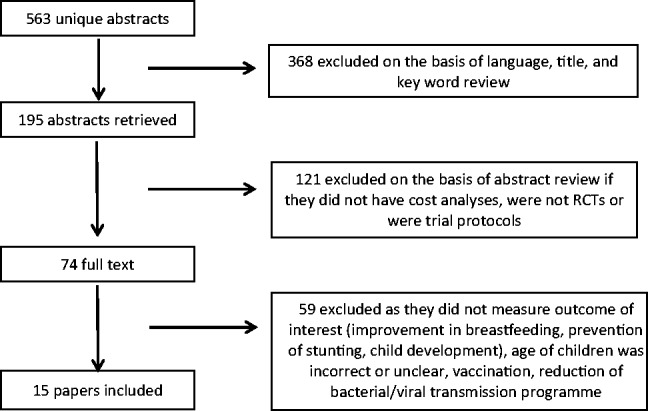
Methods of review.

The expanded search generated 563 possible articles as shown in Figure 1. Titles and key words were reviewed as a first check and 368 articles were excluded on this basis. The abstracts were reviewed and a further 121 articles were excluded. The full texts for remaining articles were then reviewed and 59 articles were excluded. This left 15 articles, which are included in this review (Tables 1, 2 and 3).

**Table 1:** Cost-effectiveness of breastfeeding interventions

Intervention	Authors	Country	Analysis/perspective	Outcome
Promoting exclusive breastfeeding	Desmond *et al.* (2008)	South Africa	Cost of delivery and CEA/provider	Cost of implementation: $1.2–27.9 million
				Cost per supported month of exclusive breastfeeding: $15.8–$84.5
				Cost per increased month of exclusive breastfeeding: $19.4–$180.6
Breastfeeding support through enhanced staff contact at a hospital	Rice *et al.* (2010)	United Kingdom	CEA/provider	Cost per QALY gained: $8951–$56 298
Peer-based breastfeeding support	Chola *et al.*(2011)	Uganda	Cost of delivery/provider	Cost per mother counselled: $139
				Cost per visit: $26
Telephone-based breastfeeding support	Hoddinot *et al.* (2012)	United Kingdom	Cost of delivery/provider	Cost per woman for proactive calls: $66.5
				Cost per woman for reactive calls: $34.1

**Table 2:** Cost-effectiveness of nutrition interventions

Intervention	Authors	Country	Analysis/perspective	Outcome
Deworming treatment to improve nutrition	Awasthi *et al.* (2000)	India	CEA/societal	Incremental cost-effectiveness ratio (ICER) per case of stunting prevented: $34.67
Home-fortification programme using zinc, iron and other micronutrients	Sharieff *et al.* (2006)	Pakistan	CEA/provider	Total cost per sachet of micronutrients: $0.02
				Cost per death averted by micronutrients: $406
				Cost per DALY averted by micronutrients: $12.2
Nutrition education programme based at health facilities	Waters *et al.* (2006)	Peru	CEA/societal	Marginal cost per case of stunting averted: $55.16
				Marginal cost per death averted: $1952
Community-based management of acute malnutrition (CMAM)	Wilford *et al.* (2012)	Malawi	CEA/provider	ICER of CMAM per DALY averted: $42

**Table 3:** Cost-effectiveness of parenting interventions

Intervention	Authors	Country	Analysis/ perspective	Outcome
Home-based parenting programme	Muntz *et al.* (2004)	United Kingdom	CEA/societal	ICER per unit improvement in T-scale of the child behaviour checklist: $361.3
Group parenting programme delivered by sure start	Edwards *et al.* (2007)	United Kingdom	CEA/provider	ICER per one point change in the Eyberg intensity score: $116.1
Home-based parenting programme	Barlow *et al.* (2007)	United Kingdom	Cost of delivery/provider	Cost of provision, intervention arm: $11 439
				Cost of provision, control arm: $6248.4
Home-based parenting programme	McIntosh *et al.* (2009). Follow-up of Barlow *et al.* (2007)	United Kingdom	Cost of delivery and CEA/societal	Mean health service costs, intervention: $9169.4
				Mean health service costs, control: $5361.3
				Cost of unit increase in maternal sensitivity: $4392
				Cost of unit increase in infant co-operativeness: $3279
Incredible years parenting programme	Bywater *et al.* (2011)	United Kingdom	Cost of delivery/provider	Cost of delivery per carer: $2808
				Mean cost per child: $6148.4
Incredible years parenting programme	O’Neill *et al.* (2011)	Ireland	CEA/provider	ICER per one point change in the Eyberg intensity score: $87
Evidence-based parenting programme	Bonin *et al.* (2011)	United Kingdom	Cost of delivery and cost savings/provider and societal	Intervention cost per family: $1535–$3351
				Cost saving to society over 25 years per family: $26 508

In the articles identified for review, costs were measured in different currencies. To facilitate comparison with World Health Organization (WHO) cost-effectiveness thresholds where appropriate, we converted all costs to International Dollars at 2005 prices. We present these figures in the text and Tables 1, 2 and 3, enabling us to examine the results in more detail. The WHO cost-effectiveness thresholds may only be applied to CEAs where the cost-effectiveness ratio is expressed as the cost per DALY averted. These thresholds classify interventions as ‘very cost-effective’ if the cost per DALY averted is less than the gross domestic product (GDP) per capita of the associated region; ‘cost effective’ if the cost per DALY averted is one to three times the GDP per capita of the associated region; or ‘not cost effective’ if the cost per DALY averted is more than three times the GDP per capita of the associated region.

We also assessed the analytical features of the included CEAs. We adapted existing guidelines, checklists and other reviews of economic evaluations of health interventions to create a questionnaire that would capture the main aspects of a CEA, with respect to the nature of ECND interventions (Drummond and Jefferson 1996; Evers *et al.* 2005; Lee *et al.* 2005; H Haghparast-Bidgoli *et al.*, unpublished data). The main economic evaluation features included in the questionnaire are presented in Table 4.

**Table 4:** Economic and methodological features of the analyses

Feature	*N*	%
Funding sources disclosed	15/15	100.00
Generalizability of findings	2/15	13.33
Sensitivity analysis performed	7/10	70.00
Outcome is discounted	4/10	40.00
Costs are discounted	6/10	60.00
ICER calculated and reported	4/10	40.00
Sources of outcome data included	13/15	86.67
Sources of cost data included	14/15	93.33
All included costs measured appropriately	9/15	60.00
All included costs valued appropriately	9/15	60.00
Important and relevant costs for alternative specified	12/15	80.00
Perspective specified	15/15	100.00
Time horizon stated	3/10	30.00
Primary objective is economic evaluation	11/15	73.33
Competing objectives clearly described	4/4	100.00

## Results

The systematic literature search identified 15 articles describing 14 interventions. Two articles performed economic evaluations of the same intervention. Seven of the articles reported the effectiveness of the intervention along with the cost-effectiveness. For the remaining eight articles, estimates of effectiveness of the interventions were published elsewhere. The identified articles spanned two broad categories of interventions: nutrition-specific interventions (*n* = 8) and parenting interventions to improve early childhood development (*n* = 7). Nutrition-specific interventions were further categorized into breastfeeding interventions (*n* = 4) and nutrition supplementation or fortification interventions (*n* = 4). Of the 15 articles, the majority were set in high-income countries (*n* = 9).

### Breastfeeding interventions

Four articles analysed the cost-effectiveness of breastfeeding interventions, two set in high-income countries and two in low- and middle-income countries. The interventions promoted or supported breastfeeding through a range of support networks. The support networks were extended through home visits made by breastfeeding councillors in South Africa (Desmond *et al.* 2008); peer support in Uganda (Chola *et al.* 2011); enhanced staff contact for mothers for low-birthweight babies in the UK (Rice *et al.* 2010) and telephone-based support in Scotland (Hoddinott *et al.* 2012). The CEAs of these interventions were performed from the perspective of the provider. The results of each CEA are presented in Table 1.

It is important to note that in each of these studies, the outcome measure or denominator used to calculate the cost-effectiveness ratio was different. Desmond *et al.* (2008) reported their outcome as cost per supported month of breastfeeding, Rice *et al.* (2010) as cost per QALY gained, Chola *et al.* (2011) as cost per mother counselled and Hoddinott *et al.* (2012) as cost per woman telephoned. Further, none of the articles presented their results in terms of the cost per DALY averted. As a result, no robust comparison of cost-effectiveness between interventions or to cost-effectiveness thresholds was possible.

### Nutrition interventions

Four articles calculated the cost-effectiveness of nutrition interventions, all set in low- and middle-income countries. The range of interventions included deworming in India (Awasthi *et al.* 2000); nutrition fortification in Pakistan (Sharief *et al.* 2006); nutrition education in Peru (Waters *et al.* 2006) and community management of severe acute malnutrition in Malawi (Wilford *et al.* 2012). The CEAs conducted by Sharief *et al.* (2006) and Wilford *et al.* (2012) were conducted from the perspective of the provider, whereas those by Awasthi *et al.* (2000) and Waters *et al.* (2006) were conducted from a societal perspective. The results of each CEA are presented in Table 2.

As with the CEAs of the breastfeeding interventions, the denominators used to calculate cost-effectiveness of these interventions were not the same. Two articles used DALYs averted (Sharief *et al.* 2006; Wilford *et al. *2012) and two used the number of cases of stunting averted (Awasthi *et al.* 2000; Waters *et al.* 2006). Although it was not possible to compare the cost-effectiveness reported in all four articles, it was possible to compare cost-effectiveness of the pairs with the same denominator. These comparisons suggest that the home-fortification of food (Sharief *et al.* 2006) was more cost-effective than community-based management of acute malnutrition (Wilford *et al.* 2012). Similarly, deworming (Awasthi *et al.* 2000) was more cost-effective than the facility-based nutrition intervention (Waters *et al.* 2006).

As Wilford *et al.* (2012) and Sharief *et al.* (2006) present their results in terms of the cost per DALY averted, it is possible to compare them with the WHO thresholds for cost-effectiveness. Both these interventions have a cost per DALY averted that is less than the GDP per capita of the associated region, thus, classifying both interventions as very cost-effective.

### Parenting interventions

Seven articles presented the costs of parenting interventions, all set in high-income countries. No CEAs or even rudimentary costings of parenting interventions in middle- or low-income countries were found. The majority of these interventions were aimed at improving conduct disorder among children. The interventions were home-visit or practice-based. Five articles replicated or adapted previously established parenting interventions such as the Incredible Years parenting programme (Edwards *et al.* 2007; Bywater *et al.* 2011; O’Neill *et al.* 2011); and the Family Partnership programme (Barlow *et al.* 2007; McIntosh *et al.* 2009). One article evaluated a home-based parenting programme targeted at children with severe behavioural problems (Muntz *et al.* 2004). One article was a modelling exercise (Bonin *et al.* 2011). Five CEAs were conducted from the provider perspective (Muntz *et al.* 2004; Edwards *et al.* 2007; Barlow *et al.* 2007; Bywater *et al.* 2011; O’Neill *et al.* 2011) and two from a societal perspective (McIntosh *et al.* 2009; Bonin *et al.* 2011). The individual findings of these CEAs are summarized in Table 3.

Three of the seven articles conducted only a cost of delivery analysis (Barlow *et al.* 2007; Bonin *et al.* 2011; Bywater *et al.* 2011). The remaining four articles conducted CEAs but as seen in the case of the CEAs of the breastfeeding and nutrition interventions, the denominators used to calculate cost-effectiveness were not the same. Two articles used the improvement in the Eyberg intensity score (Edwards *et al.* 2007; O’Neill *et al.* 2011); one, the improvement in the T-Scale of the child behaviour checklist (Muntz *et al.* 2004) and another, the increase in maternal sensitivity and infant co-operativeness (McIntosh *et al.* 2009). Thus, it was only possible to compare cost-effectiveness of the pair with the same denominator. This comparison suggested that the Incredible Years parenting programme (O’Neill *et al.* 2011) was more cost-effective than the group-parenting programme delivered by Sure Start (Edwards *et al.* 2007). As none of the articles presented their results in terms of the cost per DALY averted, it was not possible to compare them with the WHO thresholds for cost-effectiveness.

### Features of analyses

This article reviewed five intervention costings that did not measure cost per effect and 10 CEAs. As shown in Table 4, 13 articles conducted a CEA or costing within a randomized controlled trial and two employed a modelling approach. All analyses specified their perspective. The health care provider was the most frequently adopted perspective (73%), while the remainder adopted a societal perspective.

The three main steps of costing require the identification of relevant cost items for each intervention, the measurement of resources used (in their physical units) and the proper valuation of these resources (by their prices) (Drummond and Jefferson 1996; H Haghparast-Bidgoli *et al.*, unpublished data; Batura *et al.* 2014). The majority (80%) of analyses reviewed identified all costs relevant to the intervention and perspective taken. However, several analyses (56%) did not clearly describe how they measured and valued their cost components. The majority of analyses discounted costs (60%). However, fewer discounted the outcomes (40%). Discount rates for costs and outcomes ranged from 3 to 3.5%.

The sources of cost and outcome data were clearly specified in the majority of the analyses (93% for costs and 87% for outcomes). Of these analyses, the majority of the outcome data was collected during the study (83%) while the majority of the cost data was secondary data (71%). All analyses included direct medical costs. Only one analysis estimated the cost savings to society in the long term and no studies estimated productivity losses or indirect costs.

Only three of the reviewed articles used composite outcome measures—one used QALYs (Rice *et al.* 2010) and two used DALYs (Sharieff *et al.* 2006; Wilford *et al.* 2012). The majority of reviewed studies used intermediate measures or natural units, such as cases averted or patients treated. This had significant implications for the comparability of results, which will be discussed further in the next section.

The majority of reviewed studies performed a sensitivity analysis (70%); however, only 30% reported their time horizon clearly. These time horizons varied, ranging from 1 year to the lifetime of participating individuals. Less than half of reviewed studies (47%) calculated and reported incremental cost-effectiveness ratios. The remainder reported total and average costs of delivery.

Although discussing the generalizability of results to the national level, or to other settings, can be an important element of a CEA, the majority of the articles included in the review did not do so. Only 20% discussed the generalizability to the provincial or the national level, but not to other settings.

## Discussion

One of the first studies of ECND was conducted during the 1980s in Jamaica. Since then, numerous studies of ECND interventions have shown positive effects on children’s health and nutrition, cognitive development and earning potential (Glewwe *et al.* 2001; Alderman *et al.* 2005; Heckman *et al.* 2006; Maulik and Darmstadt 2009; Nores and Barnett 2010; Engle *et al.* 2011). This article aimed to summarize what is known about the cost-effectiveness of ECND interventions and the methods used to assess that cost-effectiveness.

Our review identified that cost analyses within ECND interventions gained momentum in the mid-2000s. In our review, we found 15 CEAs of ECND interventions. Four CEAs of breastfeeding interventions were conducted in high- and low-income countries, four CEAs of nutrition interventions in low- and middle-income countries and seven CEAs of parenting interventions aimed at improving children’s behaviour in high-income countries. This suggests two key gaps in the cost-effectiveness literature: the first pertains to the evidence regarding the cost-effectiveness of parenting interventions in low- and middle-income countries, and the second to the cost-effectiveness of play and stimulation interventions in any setting. This is in spite of the fact that parenting interventions, with stimulation outcomes, have been trialled in low- and middle-income countries. Two such interventions provided parents with psycho-social support to improve child development (Carneiro *et al.* 2011; Macours *et al.* 2012). The interventions had significant, positive results but no analyses of their cost-effectiveness were conducted. Similarly, although Baker-Henningham and Lopez Boo (2010) reviewed the effectiveness of various interventions to improve child development outcomes irrespective of context, there is no published record of the cost-effectiveness of these interventions. Those cost-effectiveness studies that were identified focused on the component parts of a comprehensive ECND intervention. No articles estimated the cost-effectiveness of combined interventions. Further, depending on the context in which the CEA was conducted, the features of analyses differed greatly, with only a handful of articles conducting sensitivity analyses and discussing the generalizability of the findings.

In the articles reviewed, interventions were set in different contexts, with different intervention designs and cost structures. To facilitate comparison of findings between articles, and against the WHO cost-effectiveness thresholds, costs measured in different currencies were converted to International Dollars at 2005 prices. However, the range of denominators used to calculate cost-effectiveness ratios, and the infrequent use of DALYs or other outcome measures as a common denominator, frustrated both efforts. Only three pairs of studies used the same denominators: i.e. the DALY (Sharieff *et al.* 2006; Wilford *et al.* 2012), cases of stunting averted (Awasthi *et al.* 2000; Waters *et al.* 2006) and improvement on the Eyberg intensity score (Edwards *et al.* 2007; O’Neill *et al.* 2011). As a result, we were only able to compare cost-effectiveness for these three pairs of articles. Further, we were only able to compare the cost-effectiveness results of two analyses (Sharieff *et al.* 2006; Wilford *et al.* 2012) to the WHO cost-effectiveness thresholds. Both these interventions were very cost-effective by this definition. This highlights that, although there is evidence regarding the cost-effectiveness of ECND interventions, the usefulness of that evidence is frustrated by a lack of comparability.

The inability to compare the cost-effectiveness of ECND interventions will reduce the extent to which this evidence can be used to allocate resources between health priorities. To improve comparability between programmes, researchers should consider using a common outcome measure. For example, for nutrition interventions, the use of ‘number of cases of stunting averted’ may be appropriate. This would at least allow decision makers to compare the cost-effectiveness of nutrition interventions. However, this would not facilitate comparison with other interventions that may be targeted at the same population. Comparability could be greatly improved through the use of a denominator such as the QALY or DALY, which can be applied to a wider range of interventions. Further, using the DALY also allows comparability against international cost-effectiveness thresholds. The QALY and DALY have a number of advantages and disadvantages (Anand and Hanson 1997; Sassi 2006), and neither may fully reflect the non-health impacts of ECND interventions. However, they are currently the ‘least worst’ common denominators that facilitate a comparison of cost-effectiveness of ECND interventions. Further research is required to develop a more comprehensive outcome measure that can reflect the health and non-health benefits of different ECND interventions to facilitate a more robust comparison of cost-effectiveness. In addition to improving the comparability of evidence, these findings suggest that future work may want to consider the affordability of programmes in a way that goes beyond the application of international thresholds.
